# DOES MILITARY VETERAN STATUS PREDICT COGNITIVE FUNCTIONING IN LATER LIFE?

**DOI:** 10.1093/geroni/igae098.0943

**Published:** 2024-12-31

**Authors:** Jeremy Yorgason, Lance Erickson, Christina Marini, Anica Pless Kaiser

**Affiliations:** Brigham Young University, Provo, Utah, United States; Brigham Young University, Provo, Utah, United States; Adelphi University, Garden City, New York, United States; VA Boston Medical Center, Jamaica Plain, Massachusetts, United States

## Abstract

Stressful life experiences can impact cognitive functioning in later years. Military experiences in relation to later life cognitive functioning is not well understood, although some work has explored the impact of PTSD on cognition. Using data from 777 individuals who participated in the Life and Family Legacies study, we explored whether veteran status (n = 383) was associated with multiple facets of cognitive functioning (using subscales from the Minnesota Cognitive Acuity Screen – Orientation, Attention, Delayed Word Recall, Comprehension, Repetition, Naming, Computation, and Verbal Fluency). All but 2 Veterans in the sample were male, and males and older respondents had lower cognitive functioning scores. Results indicated that Veteran status among males was not predictive of any one cognitive functioning subscale after gender and age were taken into account, yet it was predictive of overall cognitive functioning. Additional analyses among male veterans explored the impact of combat exposure and PTSD on each cognitive functioning subscale. While combat exposure was not associated with any cognitive outcome, PTSD symptoms (as measured by the PTSD checklist – the PCL-C) were negatively associated with the verbal fluency subscale and with overall cognitive functioning (b=-.05, p=.021). Our preliminary analyses suggest that Veteran status is not necessarily associated with individual subscale measures of cognitive functioning but may be related to a broader overall measure. PTSD symptoms were also an important correlate of cognitive functioning among older male Veterans. Veterans, especially those with PTSD symptoms, may be at greater risk for cognitive declines in later life.

